# Editorial: Evolving non-invasive biomarkers: chemokines and multi-omic profiling in NSCLC immunotherapy response

**DOI:** 10.3389/fonc.2026.2002791

**Published:** 2026-10-05

**Authors:** Jacobo Rogado, Elisabet González del Portillo, Diego Casado Elía, Jaime Rubio-Perez

**Affiliations:** 1 Department of Medical Oncology, Hospital Universitario de La Princesa, Madrid, Spain; 2 Department of Radiation Oncology, Hospital Universitario Rey Juan Carlos, Móstoles, Madrid, Spain; 3 Department of Medical Oncology, Hospital General de Villalba, Collado Villalba, Madrid, Spain; 4 Department of Medicine, Memorial Sloan Kettering Cancer Center, New York, NY, United States

**Keywords:** biomarker, chemokines, immunotherapy, lung cancer, non-small cell lung cancer (NSCLC)

Immune checkpoint inhibitors (ICIs) have changed the treatment of non-small cell lung cancer (NSCLC), producing durable disease control in a proportion of patients. However, treatment selection still depends largely on biomarkers that capture only part of the tumor-immune interaction. Programmed death-ligand 1 (PD-L1) expression and tumor mutational burden (TMB) remain clinically useful, but spatial and temporal heterogeneity, assay variability, and reliance on a single tissue specimen limit their predictive accuracy (1, 2). In this context, this Research Topic examines whether repeatable, non-invasive measurements can improve the assessment of response, resistance, and toxicity. Its seven articles address this question through chemokines, soluble immune checkpoints, circulating tumor DNA (ctDNA), multi-omic profiling, and quantitative imaging.

Cabezón-Gutiérrez et al. establish the biological framework in a narrative review of chemokine signatures and multi-omic biomarkers. CXCL9, CXCL10, and CXCL13 are associated with inflamed tumors, T-cell recruitment, and tertiary lymphoid structures, whereas IL-8 and CCL2 are linked to myeloid recruitment, T-cell exclusion, and resistance. The authors emphasize that these signals are most informative when evaluated together with genomic, transcriptomic, proteomic, and metabolomic data. Shen and Gao extend this approach across the liquid-biopsy spectrum, including ctDNA, circulating tumor cells, extracellular vesicles, soluble mediators, and systemic inflammatory indices. They also discuss the integration of molecular profiling with radiomics and pathomics through multimodal and explainable artificial intelligence. Both reviews support longitudinal blood-based assessment as a complement to tissue and imaging, rather than as a substitute for the spatial information they provide.

Hu et al. broaden the clinical objective beyond efficacy prediction. Their review integrates genomic, transcriptomic, epigenomic, proteomic, metabolomic, radiomic, and microbiome signals associated with primary or acquired resistance, hyperprogressive disease, and immune-related adverse events. The proposed efficacy-risk quadrant underlines a clinically relevant principle: a useful model should estimate both the probability of benefit and the probability of serious harm. Joint probability reporting, biomarker-informed mitigation, and repeated assessment during therapy may improve clinical interpretation. Nevertheless, most component signatures and artificial intelligence models remain investigational and require prospective validation.

Quantitative evidence for a readily accessible circulating analyte is provided by Rotariu et al. Their meta-analysis included 20 studies and 1,967 patients. Higher baseline soluble PD-L1 was associated with shorter progression-free survival (pooled hazard ratio [HR], 2.26; 95% confidence interval [CI], 1.77-2.87) and overall survival (HR, 2.04; 95% CI, 1.55-2.68). The median cutoff in progression-free survival analyses was approximately 90 pg/mL. However, heterogeneity in populations, treatments, sample types, assays, and thresholds precludes adoption of a universal cutoff. Prospective standardization and serial measurements are needed to distinguish prognostic information from treatment-specific prediction.

The original ctDNA study by Gonzalez Santa-Catalina et al. illustrates the additional information contained in genome structure. Baseline plasma whole-exome sequencing was performed in 37 patients with advanced NSCLC receiving first-line ICI or chemoimmunotherapy. Single-nucleotide-variant-derived TMB did not distinguish favorable and adverse outcomes, whereas copy-number signatures showed the largest differences. CN9 was enriched among patients with adverse outcomes, particularly those with PD-L1 expression of at least 50% and early progression, whereas CN21 was more prominent in the favorable high-PD-L1 group. No signature remained statistically significant after correction for multiple testing. These findings should therefore be considered hypothesis-generating evidence that ctDNA-derived chromosomal instability may add information beyond PD-L1 and TMB.

A different route to non-invasive phenotyping is presented by Qie et al. The investigators analyzed 490 patients with EGFR-mutant, T790M-negative lung adenocarcinoma after resistance to a first-generation tyrosine kinase inhibitor. A 2.5-dimensional deep-learning model used routine computed tomography images to predict response to ICI plus chemotherapy. The model achieved areas under the curve of 0.885 in the training cohort and 0.819 and 0.863 in two validation cohorts; its score also separated progression-free survival groups. The retrospective regional design, incomplete harmonization of clinical and PD-L1 data, and absence of clinical-only and combined clinical-imaging benchmarks limit immediate application. Even so, the study shows that routine imaging may capture phenotypes that merit prospective biological and clinical validation.

Dynamic monitoring in the curative-intent setting is addressed by Yu et al. Their systematic review and reconstructed individual patient data meta-analysis included nine randomized trials and 3,888 patients with resectable NSCLC. Neoadjuvant chemoimmunotherapy improved overall survival (HR, 0.74; 95% CI, 0.66-0.83), with 5-year overall survival of 65.0% versus 54.8% and 5-year event-free survival of 52.6% versus 32.7%. Across four trials with ctDNA data, chemoimmunotherapy increased the likelihood of ctDNA clearance (odds ratio, 2.79; 95% CI, 2.05-3.81). Early clearance was associated with longer event-free survival (HR, 0.37) and overall survival (HR, 0.46), and preoperative clearance retained prognostic value among patients without a pathologic complete response (HR, 0.44 for event-free survival). These findings do not establish a biomarker-guided treatment algorithm, but they show why change over time may be more informative than a single baseline value.

Overall, this Research Topic does not support the use of a universal, single-time-point biomarker. Performance depends on disease stage, molecular context, treatment, sampling time, and the endpoint to be predicted. Previous studies of peripheral immune-cell subsets and CXCL12/CXCR4 biology support the evaluation of circulating immune phenotypes in NSCLC (3, 4), although direct prospective assessment of chemokine-receptor profiles remains limited. Clinical translation will require harmonized assays, prespecified thresholds and endpoints, independent external validation, calibrated models, and comparisons with standard clinicopathologic variables. Biomarker-guided trials must then show that using the result changes management and improves patient outcomes. The most useful solution is unlikely to be the largest panel; it will be the smallest reproducible combination that provides actionable information within an acceptable cost and turnaround time.
